# The Lush Fucales Underwater Forests off the Cilento Coast: An Overlooked Mediterranean Biodiversity Hotspot

**DOI:** 10.3390/plants12071497

**Published:** 2023-03-29

**Authors:** Francesco Rendina, Annalisa Falace, Giuseppina Alongi, Maria Cristina Buia, João Neiva, Luca Appolloni, Giuliana Marletta, Giovanni Fulvio Russo

**Affiliations:** 1 Department of Science and Technology, University of Naples “Parthenope”, 80143 Naples, Italy; 2 Department of Life Sciences, University of Trieste, 34127 Trieste, Italy; 3 CoNISMa National Inter University Consortium for Marine Sciences, 00196 Roma, Italy; 4 Department of Biological, Geological and Environmental Sciences, University of Catania, 95124 Catania, Italy; 5 Zoological Station Anton Dohrn of Naples, 80077 Ischia, Italy; 6 Centro de Ciências do Mar do Algarve (CCMAR), University of Algarve, 8005-139 Faro, Portugal

**Keywords:** *Cystoseira sensu lato*, Fucales, seaweed, brown algae, MPA, Tyrrhenian Sea

## Abstract

Fucales (Phaeophyceae) are ecosystem engineers and forest-forming macroalgae whose populations are declining dramatically. In the Mediterranean Sea, *Cystoseira sensu lato (s.l.)*—encompassing the genera *Cystoseira sensu stricto*, *Ericaria*, and *Gongolaria*—is the most diverse group, and many species have been shown to be locally extinct in many areas, resulting in a shift toward structurally less complex habitats with the consequent loss of ecosystem functions and services. In this study, we report on the extensive occurrence of healthy and dense marine forests formed by Fucales in the Santa Maria di Castellabate Marine Protected Area in Cilento, Italy (Tyrrhenian Sea, Mediterranean). On a total area of 129.45 ha, 10 *Cystoseira s.l.* taxa were detected using a combined morphological and molecular approach, with an average cover of more than 70%. One of these taxa has been sequenced for the first time. These findings underline the high ecological value of this area as a hotspot of benthic biodiversity and highlight the importance of marine protected area management and regional monitoring programs to ensure the conservation of these valuable yet fragile coastal ecosystems.

## 1. Introduction

Forests of brown algae (i.e., Laminariales and Fucales) are among the most productive and valuable carbon-rich marine benthic communities, extending from the sea surface to the upper circalittoral zone on photophilic rocky substrates along temperate coasts [1,2,3]. Their distribution is controlled by various abiotic (i.e., coastal geomorphology, depth, wave exposure, and nutrient concentrations [4,5,6,7,8]) and biotic (i.e., grazing [9,10,11]) factors.

In the Mediterranean Sea, the dominant canopy-forming species belong to the genera *Cystoseira sensu lato* (*s.l*.) (i.e., *Cystoseira sensu stricto*, *Ericaria*, and *Gongolaria* [12]) and *Sargassum* C. Agardh. These two groups are the most diverse among fucalean macrophytes, and their high morphological variability has hindered taxonomic identification based exclusively on a morphological [13,14]. Indeed, molecular barcoding has been increasingly used to circumscribe and identify taxonomically difficult groups of algae with a high genetic diversity, such as *Cystoseira s.l*., for which a large cox1 reference database has recently been developed [12]. These studies are essential since issues in species identification can potentially affect biodiversity baselines and ultimately misdirect conservation focus and actions.

*Cystoseira s.l*. stands provide a number of important marine ecosystem functions and services [15], including shelter and nursery for fauna, high primary productivity and carbon sequestration, coastal protection, reduction of nutrients and turbidity, direct sources for many traditional and commercial uses, aesthetic beauty, and intrinsic evolutionary value [16]. They also play a central role in understory growth by influencing light, desiccation, water movement, and the rate of transport and deposition of suspended sediment [17].

Increasing human threats, such as coastal development, habitat degradation, pollution, maritime traffic, aquaculture, and intensive fishing, have significant impacts on the distribution and ecological status of coastal marine habitats [18,19,20,21,22]. In addition to local impacts, marine macroalgae are also likely to be affected by anthropogenic ocean warming in the near future [23,24,25,26,27,28].

In recent decades, populations of *Cystoseira s.l.* have significantly reduced their range, with dominance shifting to macroalgal species with lower intrinsic complexity (i.e., turf-forming or other ephemeral opportunistic algae) or, at worst, to urchin barrens. These shifts have led to a decline in associated biodiversity and a loss of ecosystem services [5,29,30,31,32,33,34,35,36,37,38]. Although there has been a dramatic loss of these ecosystems in many regions [31,39,40,41], in other regions losses have been limited to the most affected areas and some populations have surprisingly maintained a relatively healthy state [42,43]. Moreover, restoration actions have been taken recently to return to a pristine state and restore the “ecological status” of these ecosystems [11,28,44,45,46,47,48,49].

Given their sensitivity to anthropogenic influences, several conservation measures have been taken to preserve these species and the habitats they form. In particular, *Cystoseira s.l*. forests are listed as “of community interest” under the Habitats Directive (92/43/EEC) and are considered indicators of the environmental quality of Mediterranean coastal waters under the Water Framework Directive (2000/60/EC) (i.e., EEI and CARLIT) [5,50]. Several species are protected by the Bern Convention, recognized as priority species by the Barcelona Convention and classified as endangered by several international organizations (e.g., RAC/SPA, MedPan, IUCN). In addition, around 1231 Marine Protected Areas (MPAs) have been established in the Mediterranean Sea, which have proven effective in both biodiversity conservation and fisheries enhancement [51,52,53,54]. However, current knowledge on the effectiveness of habitat protection of *Cystoseira s.l.* by MPAs is still insufficient and data on their distribution and biodiversity along Italian coasts are still fragmented and incomplete.

On the Cilento coast (SW Italy, Mediterranean Sea), the Santa Maria di Castellabate MPA (SMC) was established in 2009. Nevertheless, there is only one study from 1971 describing the macroalgal assemblages in this area [55], while no recent data have been collected so far.

The aim of this study was to investigate the distribution and species diversity of benthic macroalgae in the SMC, with particular emphasis on the highly valuable canopy-forming *Cystoseira s.l.* species, in order to assess the effectiveness of this MPA and to provide useful information for the management and conservation of these valuable marine forests.

## 2. Materials and Methods

### 2.1. Study Area

**Figure 1:**
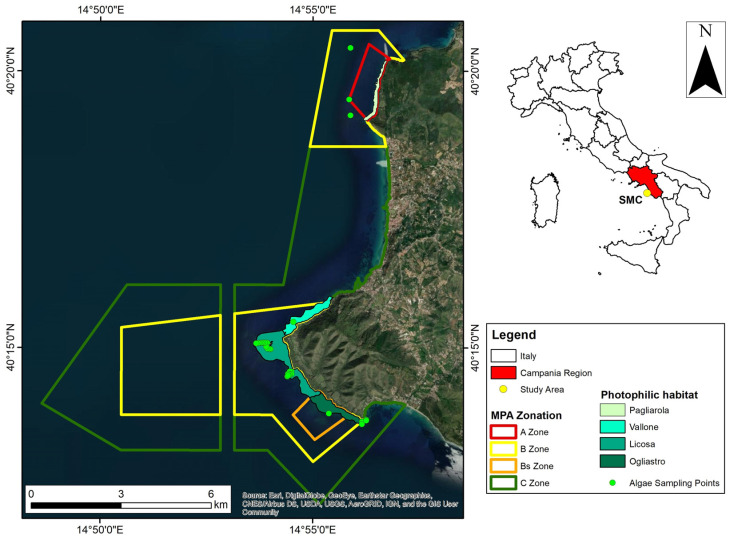
Study area in the MPA Santa Maria di Castellabate (SMC; Tyrrhenian Sea, Italy). Photophilic habitat refers to [57]. Green dots represent the algal collection sites for taxonomic identification.

The SMC (SW Italy, Mediterranean) is one of the two MPAs of the Cilento, Vallo di Diano and Alburni National Park, included since 1998 in the UNESCO World Natural Heritage for its historical, cultural, and naturalistic uniqueness, and whose ecosystems of particular floristic and faunistic richness are subject to special protection measures. The SMC extends over 7000 ha along the Cilento coast, from Punta Ogliastro to Sambuco Bay. It is divided into three protection zones (Figure 1) to balance the needs of conservation and sustainable use of resources through differentiated regulation of human activities. The C zone covers about 3750 ha [56]. It was designed as a buffer zone between the areas of greatest naturalistic value and the unprotected areas around the MPA. The B zone covers about 2750 ha. Various human activities are allowed here, but they are regulated and authorized by the managing authority to ensure sustainable use of environmental resources. The A zone (“no-take” or “integral reserve”) extends from Punta Tresino to Punta Pagliarola and was established to protect the area with the highest biological and ecological value. It represents 3% of the total protected area (170 ha). The seabed of the SMC consists of both soft and hard bottom, with sandy bays alternating with rocky coastal stretches along the coast. The latter are formed by flysches, which consist of laminar layers of different lithology formed millions of years ago by the overlaying and compaction of sediments of different grain sizes (e.g., sands, silts, and clays) that fell from the coast along the continental slope and were deposited in the abyssal plain. Due to their lithological composition, there are different types of flysch, but the most widespread in the area is called “Cilento Flysch” because of its particular layering of sandstone, clay (marl), and limestone [56]. Prior to the establishment of the MPA, a bionomic map was produced and six habitats were described [57]. The coralligenous habitat occurs in the same areas but at greater depths (>30 m), and Punta Licosa in particular has large isolated bio-concretions (i.e., coralligenous banks) scattered on the soft bottom. The “well-sorted fine sands” occur at the shallowest depths off the beaches of Santa Maria di Castellabate (between Punta Pagliarola and Punta Licosa) and off the bay of Ogliastro. *Posidonia oceanica* (L.) Delile beds grow on fine sand in water depths between 5 and 35 m. The “coarse sands and fine gravels” are mainly found off Punta Licosa and on the Tresino cliff at depths of between 25 and 50 m. The coarseness of the sediments is due to the action of the strong bottom currents that carry the finer elements offshore. The offshore “coastal terrigenous muds” are found at the boundaries of the MPA and at a depth of more than 35 m in the bay of Santa Maria di Castellabate and at a depth of more than 50 m at Punta Licosa. These muds come from the finer fractions of terrigenous sediments that enter the sea from the rivers and the numerous gullies coming down from the hills of Tresino and Licosa. Finally, the “photophilic algal communities” or “biocénose à Algues Photophiles” (AP) [58] are abundant on the peculiar flysches in the shallower part of the rocky shelf between Punta Tresino and Punta Pagliarola and in the area around Punta Licosa.

### 2.2. Field Surveys and Data Collection

**Figure 2:**
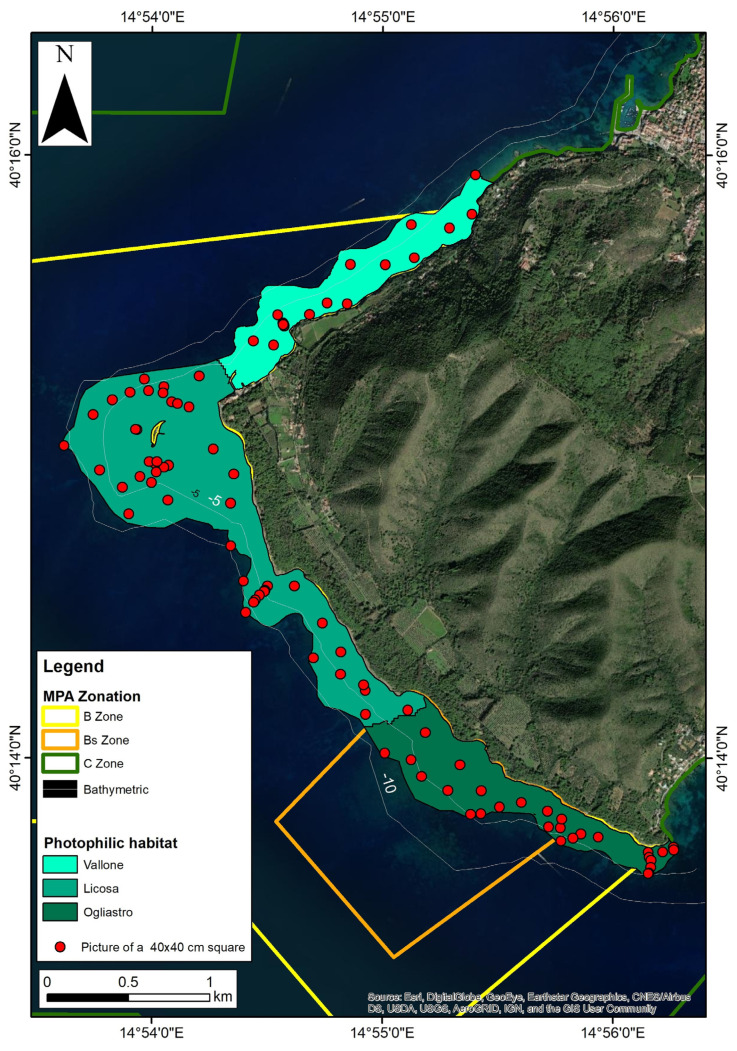
Map of photophilic habitat and spatial distribution of the 40 × 40cm quadrats/images (red dots) used for the EBK geo-statistical analysis. Photophilic habitat refers to [57]. The green scale indicates the three sites studied: Vallone, Licosa, and Ogliastro.

Surveys were conducted at SMC in June 2021, within the predicted period of maximum biomass of Fucales. In particular, the “photophilic algal habitat” identified in the bionomic map of Russo et al. [57] was surveyed (Figure 1). This habitat extends from the intertidal zone to the upper limit of the Posidonia bed and varies between 5 and 12 m water depth. A total of 95 photographs of 40 × 40 cm quadrats were taken randomly within the SMC habitat and algal cover estimated using VidAna© software (1.0.1beta) (Figure 2). Specimens of *Cystoseira s.l.* were collected at 34 stations for identification. In addition, three deep dives (ca. 30–35 m water depth) were conducted at Pagliarola outside the photophilic habitat on rocky reefs below the lower limit of *Posidonia* to determine the possible presence of *Cystoseira s.l.*

### 2.3. Genetic Identifications

Several samples of *Cystoseira s.l.* from Cilento were sequenced for the barcoding marker cox1 using the primer pairs GAZ2 [59] and cox1-789F/cox1-1378R [60] and the amplification conditions described by Neiva et al. [12]. Fragments were sequenced in a ABI PRISM 3130xl automated capillary sequencer (Applied Biosystems, Waltham, MA, USA) at CCMAR, Portugal. Sequences were aligned and proofread to previously published cox1 sequences ([12] and references therein) in Geneious Prime 2020 (http://www.geneious.com, accessed on 20 May 2022). Nomenclature follows Algaebase ([61] and references therein).

### 2.4. Geo-Statistical Analyses

To investigate the spatial distribution of the predominant algal assemblages, the data were processed to produce coverage maps using the Empirical Bayesian Kriging model (EBK). This model was chosen because it is less sensitive to low sample density. EBK differs from classical kriging methods by accounting for the error introduced by the estimation of the semivariogram model. This is done by estimating and then using many semivariogram models instead of one, and simulating the EBK with new predicted parameters until the lowest average value SE is reached [62].

In this study, EBK was performed using ArcGIS for Desktop 10.7 (Copyright © 2017, ESRI Inc., Berkeley, CA, USA) using algal cover data in the SMC B zone at the three sites characterized by complex algal patches: Ogliastro, Licosa, and Vallone (Figure 2). No EBK was performed at Pagliarola, since this site was characterized by a homogeneous photophilic habitat with no clear distribution patterns that could be mapped.

## 3. Results

### 3.1. Genetic Identifications

In total, 12 samples were successfully sequenced for a fragment of cox1, corresponding to 5 Molecular Operational Taxonomic Units (MOTUs, Table 1). Four MOTUs matched genetic entities circumscribed in previous studies, but one—*Ericaria funki*—was sequenced for the first time (Genbank sequences OQ659508-09). Three MOTUs—*Cystoseira pustulata*, *Gongolaria* sp. 2 (as *G. elegans*), *Ericaria brachicarpa*—were congruent with a priori identifications of species based on morphology. Sample CIL3A, identified morphologically as *Cystoseira crinitophylla*, was incongruent because sequence data identified it as *Ericaria crinita/barbatula* MOTU. CIL12A and CIL12B, identified as *Cystoseira* sp. were identified as *Ericaria funki*.

Therefore, the *Cystoseira s.l.* taxa found in the Cilento on a morphological and/or molecular basis are listed in Table 2, together with sampling depth and location.

### 3.2. Spatial Distribution of Photophilic Habitat

The total spatial extent of the AP habitat at SMC was 260 ha.

**Figure 3:**
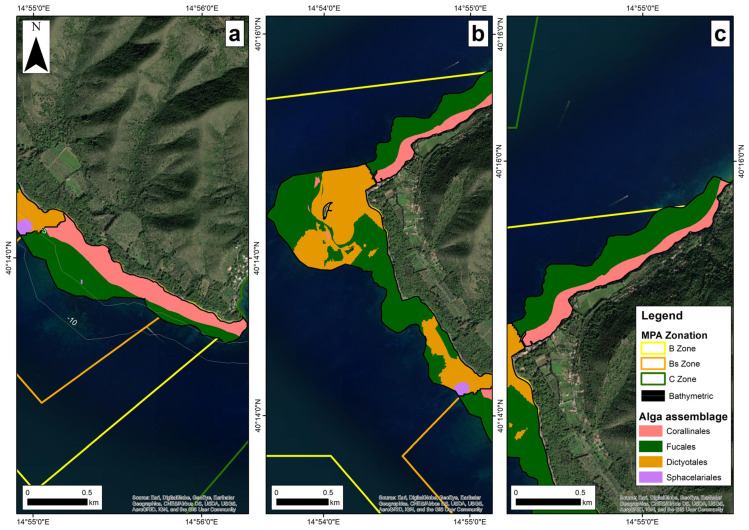
Distribution of the main algal assemblages resulting from the EBK model at the three sites of the Santa Maria di Castellabate MPA: (**a**) Ogliastro; (**b**) Licosa; (**c**) Vallone.

At Ogliastro (Figure 3a), the AP habitat covered an area of 54.63 ha. The EBK model showed that the macroalgal assemblages were dominated by Fucales (29.03 ha), extending parallel to the shoreline from 2 to 10 m depth, and Corallinales (25.55 ha) in shallow waters from 0.1 to 3 m depth; the Sphacelariales, mainly *Haloptetis scoparia* (Linnaeus) Sauvageau, (0.05 ha), were patchily scattered. The assemblage of Fucales was dominated in terms of mean percent cover by *Cystoseira s.l.* spp. (74.53 ± 28.73%), with scattered thalli of *Sargassum vulgare* C. Agardh (6.07 ± 17.32%). *Dictyota* spp. (7.13 ± 11.24%), *Corallina* sp. and *Jania* sp. (11.47 ± 15.95%), and *Padina pavonica* (0.53 ± 0.83%) were less represented. The predominant species of *Cystoseira s.l.* at this site were *Gongolaria* sp. 2 and *Cystoseira pustulata*.

At Licosa (Figure 3b), the AP habitat extended over 131.42 ha. The EBK model showed that the area was dominated by Fucales (67 ha) at a depth of 0.2 to 12 m, and Dictyotales (62.17 ha) at a depth of 0.1 to 3 m, followed by scattered Sphacelariales (1.46 ha) and Corallinales (0.61 ha). In the assemblage of Fucales, *Cystoseira s.l.* spp. accounted for the largest mean percent cover (77.48 ± 28.73%) and *Dictyota* spp., *Corallina* sp., *Jania* sp., *Neogoniolithon brassica-florida* (Harvey) Setchell and Mason, *Laurencia* sp., *P. pavonica,* and *H. scoparia* for less than 10%. Most *Cysoseira s.l.* species were encountered at this site, with *Ericaria corniculata*, often in association with *Cystoseira crinita/barbatula*, being the most abundant, especially between 0 and 6 m depth, followed by *Gongolaria* sp. 2 and *Cystoseira pustulata*, which were principally found in deeper waters (5–12 m).

At Vallone (Figure 3c), the AP habitat covered 49.3 ha. The EBK model showed that the macroalgal assemblages were dominated by Fucales (33.42 ha) along an almost continuous belt between 2 and 8 m depth, above which the Corallinales belt (15.87 ha) developed between 0.2 and 2 m depth. The Dictyotales (0.01 ha) formed small patches. The Fucales assemblage was dominated by *Cystoseira s.l.* spp. with an average percent cover of 90.25 ± 4.69%. Other taxa, but with an average cover of less than 6%, were *Dictyota* spp., *Corallina* sp., *Jania* sp., and *P. pavonica*. The only species of *Cystoseira s.l.* found at the site was *Ericaria corniculata*.

At Pagliarola, the AP habitat covered 25.2 ha. It consisted of Sphacelariales and Dictyotales. No Fucales were found at depths between 0 and 12 m, although three deep species *Ericaria funkii*, *E. zosteroides,* and *Gongolaria montagnei* were collected at depths of 30–35 m on rocky cliffs below the lower limit of the *P. oceanica* bed.

## 4. Discussion

In a general scenario where populations of *Cystoseira s.l*. are rapidly declining along Mediterranean coasts [31,40,41,42,63,64,65], leading to a shift toward structurally less complex habitats with a depletion of biodiversity, ecosystem functions, and services, the SMC MPA with its lush and extensive *Cystoseira s.l.* forests is a striking exception for the Italian coasts.

**Figure 4:**
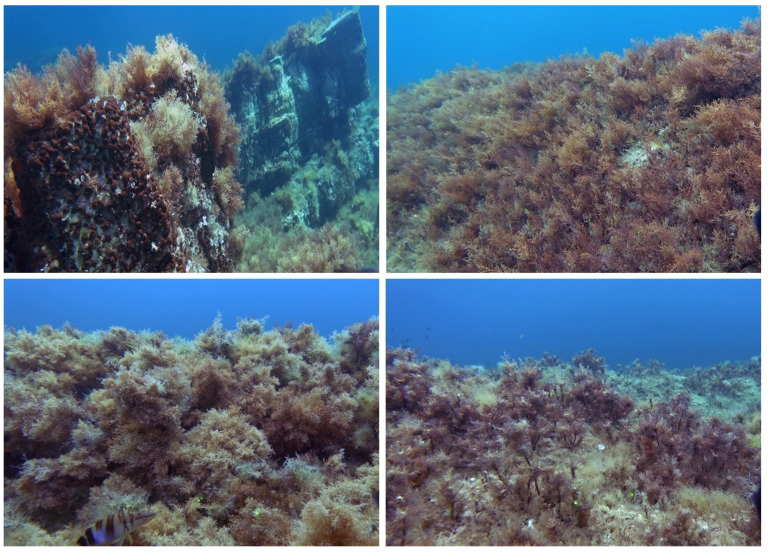
*Cystoseira s.l.* forests covering the Cilento Flysh of the Santa Maria di Castellabate MPA.

We report extensive areas totaling 129.45 ha dominated by Fucales with an average cover of more than 70%. We also found a large number of Fucales, with 10 *Cystoseira s.l.* taxa (Table 2) and *Sargassum vulgare*. These findings are probably related to the low impact of anthropogenic activities in the first meters of depth within the MPA, the oligotrophic waters and the geomorphology of the bottoms. Indeed, this area is very peculiar for the occurrence of flysches. They could have represented an optimal substrate for extensive colonization by such a large number of Fucales due to their variable lithological composition/porosity, relative flatness, and bottom morphology characterized by ridges, 30–40° inclined, parallel with one another starting from the coastline (Figure 4).

Edwards et al. [55] already reported a high coverage of Fucales in the SMC in 1971. In particular, in the shallower intertidal, between 0 and 0.5 m depth, *C. foeniculacea* [as *C. discors* (L.) C. Agardh], *C. compressa* [as *C. fimbriata* (Desf.) Bory], and *Ericaria crinita* (Duby) Molinari and Guiry [as *C. crinita* (Desf.) Bory]; in the subtidal, between 0.5 and 10 m depth, above the *Posidonia* bed, the predominant taxa were *C. jabukae* Erceg, *G. montagnei* [as *C. spinosa* Sauv.], three unidentified *Cystoseira* and *Sargassum vulgare*. Some differences in species composition were noted in our study (see Table 2), partly due to misidentifications and taxonomic reassessments. In particular, it is worth noting that, according to Verlaque et al. [66], all previous records of *C. jabukae* from the western Mediterranean and the southern Tyrrhenian Sea are probably related to *E. funkii*. This species was sequenced here for the first time, confirming its close affinity with the *Ericaria selaginoides/amentacea* species complex. Our sequence data revealed some cases of taxonomic conflict within *Ericaria*. In particular, the taxonomic species “CIL1A” (*Cystoseira* sp.) and “CIL3A (*Cystoseira crinitophylla*) (in Table 1) were respectively assigned to *E. brachycarpa* and *E. crinita/barbatula* MOTUs based on previously published sequence data. Taxonomic conflicts within the *Ericaria crinita* lineage (which also includes *E. barbatula*, *E. giacconei*, *E. corniculata*, *E. brachycarpa, E. balearica,* and *E. crinitophylla*) have been raised in recent taxonomic studies based on morphological, sequence and species delimitation methods [12,67,68], pinpointing the taxonomic difficulties of the group. Samples of *Gongolaria elegans* were also recovered as belonging to *Gongolaria* sp. 2, a genetic entity previously documented from the eastern Mediterranean and Sicily [12] and whose range has now been extended also to the southern Tyrrhenian Sea. Ongoing work will further clarify the identity and affinities of the *Cystoseira s.l.* forests of Cilento.

Furthermore, it is worth noting that in 1971 *Cystoseira s.l.* was present at Pagliarola [55], whereas no shallow Fucales were observed in our study. The disappearance of these endangered species at this site is likely due to its location between the two towns of Agropoli and Santa Maria di Castellabate, which experienced heavy urban development in the 1970s, leading to habitat destruction and water pollution. However, the construction of new buildings is nowadays highly regulated/restricted and most human activities affecting the Pagliarola site (e.g., fishing) have been reduced owing to the establishment of the A zone of the terrestrial National Park (1991) and of the more recent MPA (2009). Therefore, the reintroduction of these species should be feasible through effective restoration measures [44,46,48].

In our study, we also noted the association of *E. corniculata* and *C. crinita/barbatula*. In Italy, *E. corniculata* occurs mainly in the Ionian and Adriatic Seas, is rare in the upper/middle Tyrrhenian Sea, and is doubtful in Sardinia, being confused with specimens of other small and slender *Cystoseira s.l*. species (www.specieaspim.it/aspim/specie, accessed on 20 February 2023). In the SMC, we found a particular distribution of these two species along the top of the flysch ridges, where *E. corniculata* grows on the flysch borders, characterized by higher hydrodynamics, and *C. crinita/barbatula* occurs in the innermost part of the flysch ridges, where wave energy is lower.

We also found deep-water assemblages (below 30 m) of *Cystoseira s.l*. species, i.e., *G. montagnei, E. funkii,* and *E. zosteroides*, growing on rocky substrates colonized by typical coralligenous taxa. Deep assemblages dominated by the coexistence of these *Cystoseira s.l*. taxa have also been found in the north-western Mediterranean in the Scandola MPA (France) at a depth of 38–50 m, and in the Port-Cros National Park at a depth of 27–47 m [69,70]. These deep forests have a high species diversity and provide substrate for many epiphytes and shelter and food for many invertebrates and fish [70]. *G. montagnei* occurs on the coasts of the western Mediterranean at depths of 8 and 50 m, and its populations have been found to be declining in favor of populations of other brown and green algae or sea urchin barrens [71]. *E. funkii* is a widespread species in the western Mediterranean and the westernmost part of the Eastern Mediterranean [69]. *E. zosteroides* occurs along the coasts of the Western Mediterranean at depths of 15 to 50 m [72], but with an increasingly fragmented distribution due to human impact [31]. It is a long-lived species (up to 50 years) with a low dispersal ability [73] and very low mortality, recruitment, and growth rates [70]. Indeed, the growth rates of deep *Cystoseira s.l.* species such as *E. zosteroides* and *G. montagnei* (0.5–0.8 cm per year^−1^) are even lower than those of slow-growing gorgonians inhabiting the same habitats, such as *Paramuricea clavata, Eunicella singularis, Eunicella cavolinii* (0.8–2.7 cm year^−1^) ([70] and references therein).

The very low growth dynamics of these deep old *Cystoseira s.l* forests make them particularly vulnerable to anthropogenic impacts [74]. Indeed, they have already disappeared from several regions [31,75], although they still persist in some limited areas of the Mediterranean [69,70]. The reasons for their decline in several areas of the Mediterranean are probably related to changes in water turbidity and sedimentation, eutrophication, mechanical effects of mooring and bottom fishing, and ocean warming [31,70,76,77,78]. In addition, biological factors such as overgrazing by sea urchins due to overfishing or the impact of invasive turf-forming algae have also contributed to the disappearance of these assemblages [70,71]. However, these special and endangered macroalgal forests remain mostly overlooked due to their great depth, hence, comprehensive monitoring programs are urgently needed to assess their ecological status and improve their management and conservation.

## 5. Conclusions

This work unveils the presence of a biodiversity hotspot with several forest-forming *Cystoseira s.l.* species off the coast of Cilento (Italy, Tyrrhenian Sea). We reported the occurrence of ten taxa of *Cystoseira s.l.* forming extensive and healthy forests in the B zone of the MPA of Santa Maria di Castellabate, giving also the opportunity of sequencing for the first time the taxon *E. funkii*. These results are probably related to the presence of very peculiar geological formations, the Cilento flysches, on which these lush forests thrive.

These findings underline the high ecological value of this area and contrast with what has been observed in other areas of the Mediterranean, where dramatic losses of Fucales have occurred in recent decades [31,40,41]. The good conservation status of the Fucales populations of SMC MPA is probably due to the lack of habitat destruction, low eutrophication and pollution, and strict regulation of fisheries within the MPA boundaries. Only Pagliarola seems to lack *Cystoseira s.l.* compared to the unique previous study [55], although this disappearance is likely antecedent to the establishment of the National Park and the MPA and, hence, a restoration of this habitat is now a feasible option.

## Figures and Tables

**Table 1:** Genetic identifications of *Cystoseira s.l.* samples from Cilento based on *cox*1 sequences.

Sample	Genetic ID (MOTU)	Comment
CIL7A	*Cystoseira pustulata*	Confirmed species throughout the whole Mediterranean, Macaronesia
CIL8A		
CIL8B		
CIL1A	*Ericaria brachycarpa*	Confirmed at least from the central Mediterranean, apparently replaced in the western Mediterranean by *Ericaria balearica*
CIL3A	*Ericaria crinita/* *E. barbatula*	Species complex confirmed throughout the whole Mediterranean
CIL12A	*Ericaria funkii*	So far sequenced only from Cilento. Reported also from the western Med. but additional sequences required to confirm it is the same taxon
CIL12B		
CIL4A	*Gongolaria* sp. 2	*Gongolaria* sp. 2 has been commonly identified as *G. elegans*, also as *G. montagnei* or *G. squarrosa*, but represents a distinct entity confirmed from throughout the central and eastern Mediterranean. The true *G. montagnei* and *G. elegans* are apparently restricted to the western Mediterranean, where they appear to replace *Gongolaria* sp. 2 (the names apply to these western species on the basis of type material)
CIL4B		
CIL5A		
CIL9A		
CIL11A		

**Table 2:** List of *Cystoseira s.l*. taxa at the Santa Maria di Castellabate MPA. O = Ogliastro; L = Licosa; V = Vallone; P = Pagliarola.

Species	Site	Depth (m)
*Cystoseira pustulata* (Ercegovic) Neiva and Serrão	O, L	3–12
*Cystoseira compressa* (Esper) Gerloff and Nizamuddin	L	0.2–0.5
*Ericaria corniculata* (Turner) Neiva and Serrão	O, L, V	2–8
*E. crinita/barbatula* (as *Cystoseira crinitophylla*)	L	3–12
*Gongolaria* sp. 2 (as *Gongolaria elegans*)	O, L	4–12
*Ericaria brachycarpa* (J. Agardh) Molinari and Guiry	O, L, V	2–10
*Cystoseira foeniculacea* (Linnaeus) Greville	L	5–12
*Gongolaria montagnei* (J. Agardh) Kuntze	P	35
*Ericaria funkii* (Gerloff and Nizamuddin) Molinari and Guiry	P	30
*Ericaria zosteroides* (C. Agardh) Molinari and Guiry	P	35

## Data Availability

The data of this study are available from the corresponding author upon reasonable request.
